# The extracellular vesicles in HIV infection and progression: mechanisms, and theranostic implications

**DOI:** 10.3389/fbioe.2024.1376455

**Published:** 2024-04-09

**Authors:** Zhen Tang, Yao Lu, Jiu-Long Dong, Wen Wu, Jian Li

**Affiliations:** ^1^ AIDS Prevention and Control Center of Yichang Third People’s Hospital, Third People’s Hospital Affiliated to Sanxia University, Yichang, Hubei, China; ^2^ Yichang Changyang County People’s Hospital, Yichang, Hubei, China

**Keywords:** extracellular vesicles, HIV, infection, progression, mechanisms

## Abstract

Extracellular vesicles (EVs), these minute yet mighty cellular messengers are redefining our understanding of a spectrum of diseases, from cancer to cardiovascular ailments, neurodegenerative disorders, and even infectious diseases like HIV. Central to cellular communication, EVs emerge as both potent facilitators and insightful biomarkers in immune response and the trajectory of disease progression. This review ventures deep into the realm of EVs in HIV-unraveling their pivotal roles in diagnosis, disease mechanism unravelling, and therapeutic innovation. With a focus on HIV, we will highlights the transformative potential of EVs in both diagnosing and treating this formidable virus. Unveiling the intricate dance between EVs and HIV, the review aims to shed light on novel therapeutic strategies that could significantly benefit HIV therapy, potentially even leading to the eradication of HIV.

## 1 Introduction

Extracellular vesicles (EVs), formed within cells’ endosomal network, are secreted by almost all cell types and primarilycomposed of proteins, lipids, various RNA forms, and DNA, reflecting their cell of origin (Théry et al., 2002; Raposo and Stoorvogel, 2013). These vesicles facilitate crucial intercellular communication by transferring their content to other cells, thereby influencing recipient cells’ behavior. This process plays a pivotal role in immune responses, the spread of cancer and neurodegenerative diseases progression, and various physiological and pathological states (Valadi et al., 2007; Yáñez-Mó et al., 2021).

In HIV/AIDS research, EVs are noteworthy for their ability to impact viral transmission and immune responses. They are instrumental in both promoting and suppressing Human Immunodeficiency Virus (HIV) infection, thereby advancing our understanding of the disease and its treatment strategies, including vaccine development (Chen et al., 2021; Habib et al., 2023).

The presence of EVs in bodily fluids like blood, urine, and saliva makes them potential biomarkers for various diseases. Their capability to transport a wide range of molecules also makes them promising for drug delivery applications, especially for targeted therapies due to their biocompatibility and ability to cross biological barriers like the blood-brain barrier (Tkach and Théry, 2016; Kalluri and LeBleu, 2020a).

**FIGURE 1:**
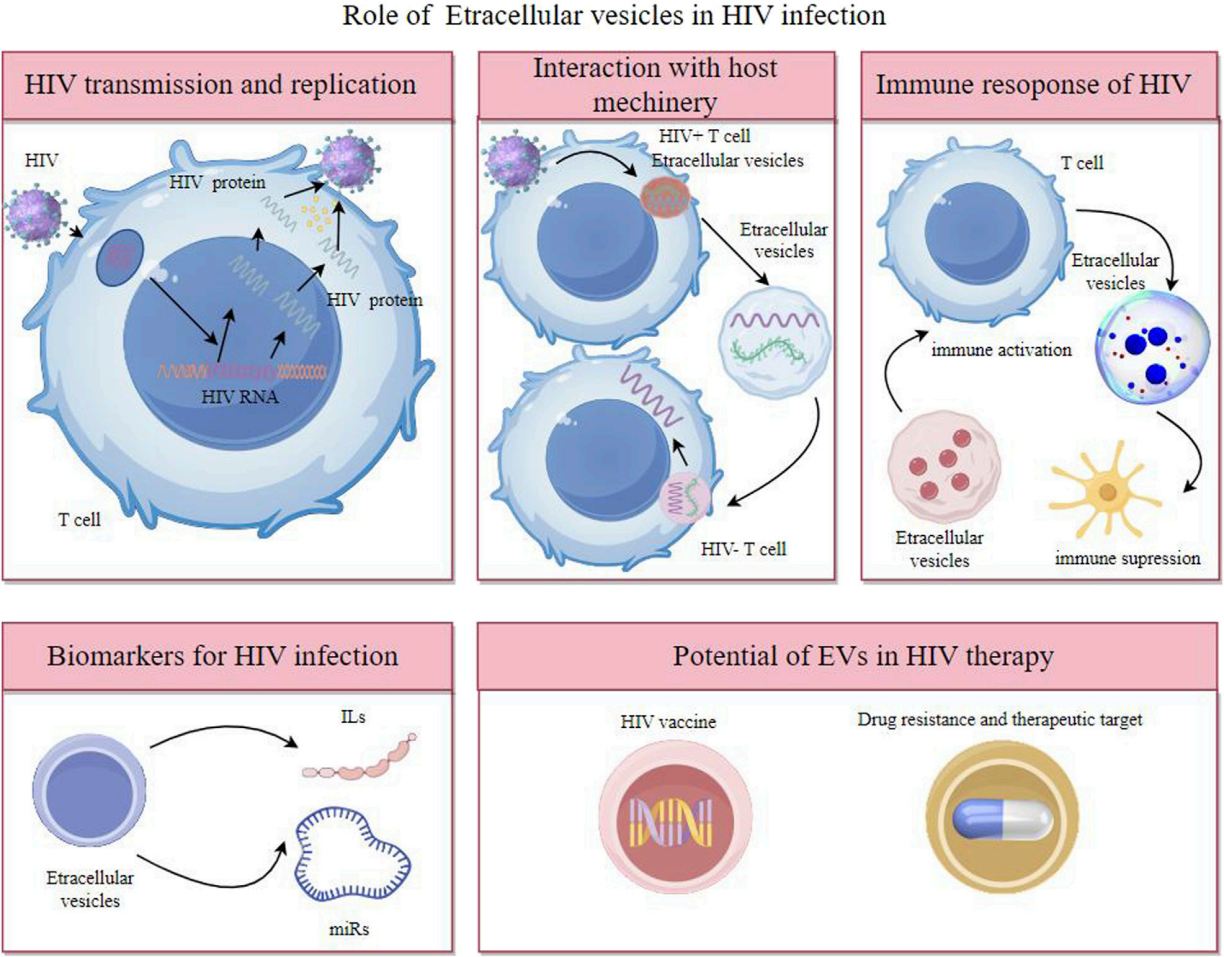
Extracellular vesicles participates in HIV transmission and replication, interaction with host machinery, immune response of HIV and have potent potential as biomarkers of diagnosis and therapeutic target.

Overall, EVs are key in intercellular communication, impacting health and disease through the transfer of molecular signals, offering insights into biological processes, and presenting novel approaches in diagnostics and therapeutics in HIV (Figure 1).

## 2 Overview of HIV infection and disease progression

HIV is a complex retrovirus that primarily attacks the human immune system, particularly CD4^+^ T cells, leading to a progressive decline in immune function. The course of HIV infection include initial infection and acute phase, asymptomatic infection stage and Acquired Immunodeficiency Syndrome (AIDS) stage. Infection begins when the virus enters the body, commonly through sexual contact, blood transfusion, or from mother to child during childbirth or breastfeeding.

**FIGURE 2:**
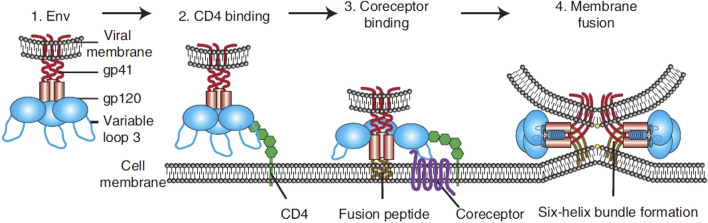
Overview of HIV entry. To transport its viral components into cells, HIV Env, made up of gp120 and gp41 units (1). Initially adheres to the host cell by attaching to CD4 (2). This action triggers structural alterations in Env, facilitating the attachment to coreceptors, significantly influenced by the V3 loop of Env (3). This step activates the membrane merging process, as the fusion peptide of gp41 integrates into the target membrane, succeeded by the creation of a six-helix bundle and the completion of membrane fusion (4) (Wilen et al., 2012).

**FIGURE 3:**
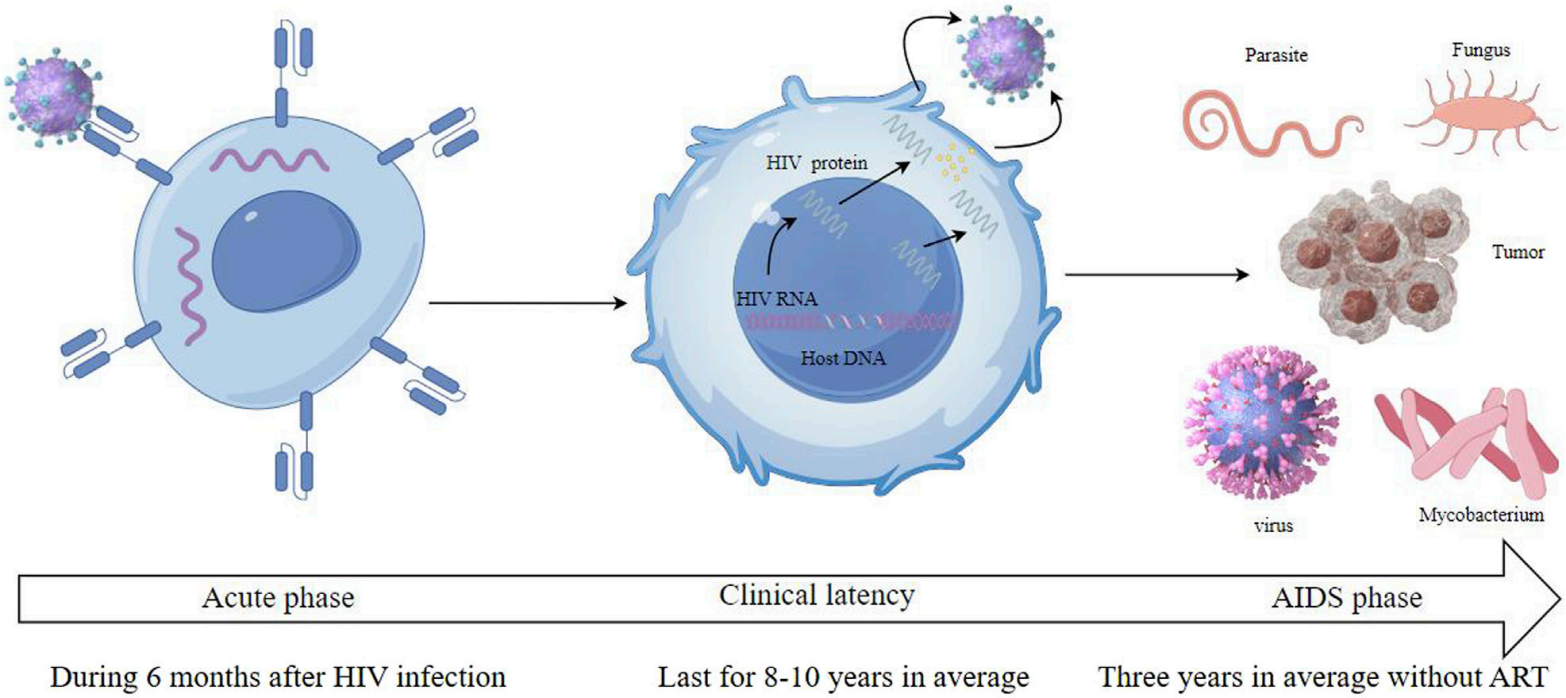
HIV infection includes three stages which are called acute phase, clinical latency and AIDS phase.

The virus binds to the CD4 receptors on the surface of T cells, integrating its genetic content into the DNA of the host cell. This acute phase, often characterized by flu-like symptoms, witnesses a rapid rise in viral quantity and a reduction in CD4^+^ T cells. However, many individuals may remain asymptomatic during this phase. Following the acute phase, HIV enters a chronic phase, often termed clinical latency or symptom-free HIV infection. In this phase, the virus persists in multiplying at reduced rates. The immune system, particularly through the action of CD8^+^ T cells, partially controls the virus, leading to a prolonged period without symptoms. Without antiretroviral therapy (ART), this phase can last for several years, but the virus remains active and continues to damage the immune system. As the disease progresses, the count of CD4^+^ T cells falls under 200 cells/mm³, which represents a severe deterioration of the immune system. Disease progress to the most advanced stage of HIV infection called as AIDS stage when the individual has one or more opportunistic infections or cancers. Common opportunistic infections include tuberculosis, pneumocystis pneumonia, toxoplasma, nontubercul-ous mycobacteria, and certain non-HIV virus and fungal infections. Kaposi’s sarcoma and non-Hodgkin lymphoma are among the cancers more prevalent in advanced HIV infection. Without treatment, the life expectancy after an AIDS diagnosis is about 3 years (Figures 2, 3).

ART has significantly transformed the outlook for individuals with HIV. By effectively reducing the viral load to undetectable levels, ART helps in the recovery of the immune system and prevents the transmission of the virus. People with HIV on ART can now expect a near-normal life span. However, ART does not cure HIV; it must be taken for life to suppress the virus. Despite significant advances in treatment, challenges in HIV management persist. These include issues related to drug resistance, side effects, and the accessibility of ART in low-resource settings. Additionally, there is an ongoing search for a cure, either through complete viral eradication or a functional cure where the virus is controlled without ART. Vaccine development is another critical area of research (Bandera et al., 2019).

HIV infection progresses through distinct phases, from acute infection to AIDS. Although ART has transformed HIV into a manageable chronic condition, challenges remain, underscoring the need for continued research and global efforts in prevention, treatment, and potential cure strategies.

## 3 The intersection of extracellular vesicles and HIV

The intersection of extracellular vesicles and HIV is a fascinating area of study, revealing complex interactions and shared pathways between these biological entities (Ouattara et al., 2018; Li et al., 2019). Their relationship is crucial in understanding HIV pathogenesis and developing new therapeutic strategies. The complex interactions between HIV and EVs focus on how HIV manipulates EVs to benefit its replication, evade the immune system, and contribute to disease progression and associated pathologies. Retroviruses and extracellular vesicles both possess a lipid makeup characterized by notably elevated amounts of cholesterol and glycosphingolipids and share enriched protein components including tetraspanins, GPI proteins, Lamps, integrins, MHC proteins, and various cytoplasmic proteins such as actin, cyclophilin, tsg101, and heat shock proteins compared to the plasma membrane (Gould et al., 2003).

Both EVs and HIV exploit similar cellular pathways for their formation and release. For instance, the biogenesis and secretion of EVs through the endosomal pathway have parallels with the assembly and release of HIV particles. HIV proteins and RNA can be encapsulated in EVs and transferred between cells, resulting in pathological outcomes (Patters and Kumar, 2018). The mechanisms involved in the selective packaging of HIV-1 RNA into EVs include the role of heterogeneous nuclear ribonucleoprotein A2/B1 (hnRNP A2/B1). This protein plays a key role in controlling the sorting of microRNAs (miRNAs) into EVs. Specifically, hnRNP A2/B1 binds to certain motifs present in some non-coding RNAs, facilitating their inclusion into EVs. This mechanism is evident in the sorting of HIV-1 TAR RNA, as the hnRNP A2/B1 binding motif is found within the TAR RNA sequence. Depletion of hnRNP A2/B1 through siRNA knockdown leads to the accumulation of HIV-1 genomic RNA within the cytoplasm, indicating its importance in RNA sorting. The study observed that the presence of TAR RNA within EVs persisted even with the addition of combination antiretroviral therapy (cART) (DeMarino et al., 2018). While both extracellular vesicles and HIV particles are enveloped structures, they have distinct molecular compositions. EVs contain a variety of host cell-derived proteins and nucleic acids, whereas HIV particles are composed of viral proteins and genetic material. EVs from HIV-infected cells can modulate the immune response, either enhancing or suppressing viral spread (Mahajan et al., 2021). They can carry viral antigens, potentially impacting the body’s immune recognition and response to HIV (Alvarez-Erviti et al., 2011). HIV can manipulate the exosomal machinery of the host cell for its own benefit (Kalluri and LeBleu, 2020b). HIV alters EV formation by hijacking cellular machinery, such as the ESCRT, leading to changes in the quantity and composition of EVs. This includes the incorporation of viral proteins like Nef and Gag, which modify EV function to support viral needs. For instance, the virus may alter the composition of EVs released from infected cells. This can lead to the modification of immune responses or the creation of a microenvironment that is more favorable for viral replication.

Besides, there are significant functional differences between HIV particles and EVs. EVs primarily function in intercellular communication, carrying molecules that influence recipient cells’ behavior. The presence of viral components in EVs has significant implications, including immune evasion by downregulating MHC-I molecules, promoting viral replication by preparing uninfected cells for infection, and contributing to HIV-associated co-morbidities through the carriage of inflammatory molecules. In contrast, HIV particles are infectious agents aiming to propagate the virus within the host. HIV also hijacks the ESCRT-III machinery that closely resembles the pathway used for EVs production and couples it to a membrane protein via the ALIX-syntenin interaction for its budding process (Kowal et al., 2014). This similarity in their biogenesis suggests potential therapeutic targets to control HIV replication (Ratajczak et al., 2016). Retrovirus originating from EVs can infect neighboring cells through EVs exchange, even when IgGs are present and effectively block the function of the viral Env (Connor et al., 1998). HIV may also transfer receptor molecules like C-C chemokine receptor type 5 (CCR5) through EVs to alter the expression of viral receptors on the cell surface (Mack et al., 2000).

**FIGURE 4:**
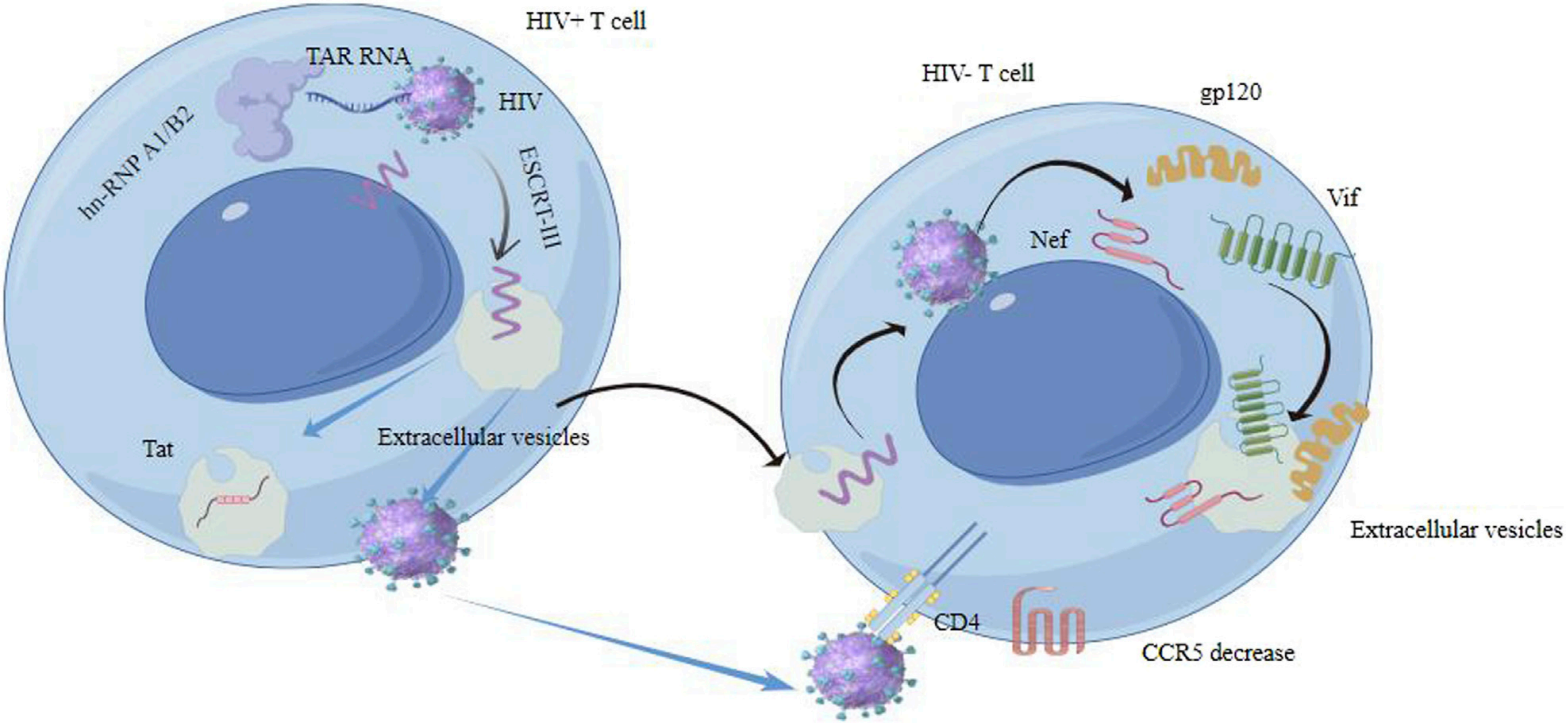
Extracellular vesicles interact with HIV contributes to HIV transmission and replication in some ways.

EVs from HIV-infected cells may enhance infection, while those from uninfected cells might have protective properties. EVs play a crucial role in HIV transmission and replication through impact on HIV lifecycle, modulation of immune response and HIV latency and reactivation (Liu et al., 2020). They can impact various stages of the HIV lifecycle and carry viral proteins and RNA, influencing the virus’s ability to infect new cells and replicate (Zhang et al., 2020; Kose et al., 2022). EVs released from HIV-1-infected cells can induce quiescent CD4^+^ T lymphocytes to become permissive to HIV-1 replication through mechanisms dependent on HIV-1 Nef protein and the ADAM17 enzyme. The ADAM17 enzyme is responsible for converting pro-tumor necrosis factor alpha (pro-TNF-α) into its active form (Arenaccio et al., 2014). Meanwhile, Nef, a protein from the HIV, facilitates viral replication and the advancement of AIDS. It affects signaling within cells and plays a role in the release of extracellular vesicles. Nef expression enhances the secretion of EVs, which can penetrate uninfected CD4 T cells, resulting in their apoptosis. Three key mRNAs (AATK, SLC27A1, and CDKAL) in extracellular vesicles from Nef-expressing cells are vital for apoptosis and fatty acid transport (Kaddour et al., 2020). The secretion of exosomal Nef (exNef) as well as protein trafficking is influenced by a specific motif in Nef, the secretion modification region which is hypothesized to bind a cellular protein (Shelton et al., 2012). Tat protein from astrocytes and Nef mRNA can induce neuron death and increase beta-amyloid secretion, impacting neurocognitive functions. Exosomal Nef affects the blood-brain barrier and induces cytokine and chemokine secretion from microglial cells (Sadri Nahand et al., 2020) (Figure 4).

EVs from HIV-infected cells can carry viral proteins and genetic material. For example, they might contain HIV-1 Nef protein, which is known to play a role in pathogenesis by modulating immune responses and enhancing viral replication and infectivity (Chen et al., 2018). They can carry factors that influence the latent reservoirs of HIV, affecting the virus’s ability to persist in the host despite antiretroviral therapy (Imran et al., 2016). This persistence could be attributed to alterations in hnRNP A2/B1 levels. It was found that the addition of antiretroviral drugs resulted in a notable rise in the concentration of the hnRNP A2/B1 protein within EVs released by HIV-1-infected cells. A densitometry analysis indicated a significant increase in hnRNP A2/B1 expression with different titers of these drugs, suggesting that hnRNP A2/B1 may play a role in transporting short TAR RNA sequences into nascent EVs. In addition, TAR RNA-containing EVs from HIV-infected T cells are known to foster growth and progression of specific Non-AIDS Defining Cancers (NADCs) by triggering the ERK signaling pathway in a mechanism that relies on EGFR/TLR3 interaction (van Heuvel et al., 2022). The viral protein Tat targets PTEN, up-regulating miR-21 and miR-222, which contribute to apoptosis resistance in HIV-1 infected CD4^+^ T cells (Sánchez-Del Cojo et al., 2017). Multiple miRNAs affect HIV replication and activity. MiR-29a, miR-128, miR-28, and miR-223 can decrease HIV replication by targeting different regions of the HIV genome (Tsai and Yu, 2010). Anti-HIV-1 miRNAs like miR-29a, miR-29b, miR-149, miR-378, and miR-324-5p inhibit HIV-1 activity by targeting auxiliary receptors or directly through genes of the HIV-1 genome. The 3′UTR region of HIV-1 RNA genome is a target of certain miRNAs, which can activate latent HIV-1, potentially leading to clearance of latent viral reservoirs (Huang et al., 2007a). Hussein et al. found that semen extracellular vesicles contain a diverse population abundant in mRNA that codes for tetraspanin exosomal markers and multiple antiviral agents. Upon being taken up by recipient cells, these EVs effectively suppress the replication of a diverse range of HIV-1 strains following their internalization. Multiple interactions occure between ANXA4, CCT6A, their paralogs, and HIV proteins. For example, gp120, a glycoprotein on the HIV surface, can upregulate ANXA4. The HIV Tat protein can upregulate CCT6A. The HIV Vif protein interacts with CCT6A. The protein-nucleic acid binding activity of these DNA binding factors in semen EVs is maintained irrespective of HIV infection and plays a role in regulating both host and HIV transcription. The anti-HIV-1 activity of semen EVs is specific to retroviruses, as evidenced by their ability to block replication of LP-BM5 murine AIDS virus complex (Connor et al., 1998).

EVs can modulate the immune response in the context of HIV infection. They can either activate immune responses against the virus or, conversely, contribute to immune suppression. This dual role is complex and depends on various factors, including the stage of HIV infection and the cell type from which the EVs originate. The intersection of EVs and HIV involves shared cellular pathways, distinct properties, and a complex interplay in the transmission and replication of the virus. Understanding these interactions is key to unraveling HIV pathogenesis and could lead to novel approaches in managing and treating HIV infection (Figure 4).

### 3.1 Extracellular vesicles in HIV disease progression

EVs play a multifaceted role in the progression of HIV disease, influencing various aspects of the virus’s pathogenesis, impacting latency and chronic inflammation, and serving as carriers of HIV-related biomarkers. Their involvement offers insights into the complex dynamics of HIV infection and potential avenues for therapeutic intervention.

The mechanisms of exosome-mediated HIV pathogenesis involve intricate interactions between the virus (Wang et al., 2020a), the host cell’s exosomal pathways (Esfandyari et al., 2021), and the immune system. Lymphocytes, macrophages, and immature dendritic cells actively exchange EVs during immune surveillance and signaling. This implies that these immune cells possess an inherent, minimal vulnerability to retroviral infection through the exchange of exosomes. This susceptibility is increased by the movement of immune cells throughout the body as part of immune surveillance activities and transmission between individuals during activities like sex and breastfeeding (Li et al., 2019). The presence of fibronectin and galectin-3, which are not found in EVs derived from T cells. These molecules are crucial for the exosome-mediated transmission of HIV-1 to T cells (Kulkarni and Prasad, 2017a). Certain contents of EVs, like microRNAs (miRNAs) and other non-coding RNAs, can carry viral components and host cell molecules that either promote or inhibit HIV replication. For example, EVs from infected cells can contain viral proteins or RNA that facilitate the infection of new cells (Wang et al., 2021). HIV can modify the contents of extracellular vesicles secreted by infected cells. These EVs may contain viral RNA, proteins like Nef (Gould et al., 2003) and Gag (Kulkarni and Prasad, 2017a), and other modified host molecules. This alteration can impact how these EVs interact with other cells and modulate the immune response (Ellwanger et al., 2016). Meanwhile, EVs can alter the microenvironment of host cells, creating conditions favorable for HIV infection and replication (Sampey et al., 2016a; Yuan et al., 2019). This includes modulating immune responses or inducing changes in neighboring cells that facilitate viral spread (Li et al., 2008). HIV-1 can become “entrapped” in EVs aggregates. This entrapment allows the virus to hitch a ride with the EVs and enhance its ability to infect new cells and bind to PtdSer receptors such as TIMs (including TIM-4) by phosphatidylserine (PtdSer) rich in surface of HIV-1 (Sims et al., 2017), thereby facilitating its dissemination to other cells. The infection facilitated by microvesicles (MVs) and EVs is affected by various cell surface receptors and adhesion proteins. These interactions are likely critical for the entry of the virus into new host cells, especially macrophages in which HIV completes its life cycle successfully but not in CD4 negative cells (Kadiu et al., 2012). Impact of EVs on HIV latency a state where the virus remains dormant in infected cells and chronic inflammation is by means of molecules carried by EVs affecting the activation or repression of latent HIV and impacting the reservoirs of the virus in the body (Yoshioka et al., 2014). In HIV-infected individuals, chronic inflammation is a significant issue, even in those receiving antiretroviral therapy. EVs can contribute to this inflammation by carrying inflammatory cytokines or by activating immune cells in a manner that perpetuates a state of chronic immune activation (Barile and Vassalli, 2017). EVs from infected cells can facilitate the spread of HIV. They can transfer viral components to uninfected cells, potentially making these cells more susceptible to infection (Fais et al., 2016). This process can occur even in the absence of direct cell-to-cell contact. They can also induce apoptosis in uninfected bystander cells. This mechanism is particularly relevant in the depletion of CD4^+^ T cells in HIV-infected individuals, contributing to immunodeficiency.

Conversely, EVs can also suppress immune responses, aiding the virus in evading immune detection. For instance, the negative regulatory factor (Nef) protein found in EVs can downregulate MHC-I molecules on the surface of target cells, impairing the ability of cytotoxic T lymphocytes to recognize and kill infected cells (El Andaloussi et al., 2013). The immune environment is modulated in ways that might affect co-infections (like tuberculosis) and other complications in HIV-infected individuals.

As carriers of HIV-related biomarkers, EVs have powerful potential in diagnosis, monitoring response to therapy and predicting disease progression. HIV-related genetic material and proteins can be encapsulated and transported by EVs which makes them potential biomarkers for diagnosing HIV infection or monitoring disease progression (de Carvalho et al., 2014; Anel et al., 2019). The composition of EVs may change in response to antiretroviral therapy, offering a window into the effectiveness of treatment and the dynamics of the viral reservoir (Ellwanger et al., 2017; Mukhamedova et al., 2019). The molecular contents of EVs might provide insights into the likely course of HIV infection in an individual, predicting faster progression or potential complications.

EVs have a significant yet complex role in HIV disease progression. They are involved in mechanisms that either facilitate or hinder the pathogenesis of HIV, impact the crucial issue of viral latency and chronic inflammation, and offer potential as carriers of biomarkers for HIV infection and its management.

### 3.2 Extracellular vesicles in immune response of HIV

EVs are identified as key players in immunomodulation involving in antigen presentation, immune tolerance, immune suppression and immune activation. Extracellular vesicles from CD4^+^ and CD8^+^ T-cells can influence dendritic cells (DCs), leading to antigen-specific T-cell dormancy. Regulatory T-cells secrete EVs that initiate immune suppression and inhibit Th1 immune responses. Extracellular vesicles expressing CD73 from regulatory T-cells can inhibit the stimulation of CD4^+^ T-cells. Extracellular vesicles, which affect both immune activation and suppression, modulating T-cell responses (Hernández-Walias FJ. et al., 2020; Campbell and Mocchetti, 2021), play a pivotal role in the immune modulation of HIV infection. Immune responses are activated by EVs presenting antigens to immune cells, thereby initiating an adaptive immune response by molecules such as MHC-peptide complexes, costimulatory molecules, and various cytokines (Sun et al., 2017). The humoral response to viruses typically involves the production of subtype G immunoglobulins (IgG) that are responsible for blocking the function of the viral envelope protein (Env) and neutralizing the virus. This neutralization prevents the virus from infecting host cells. Chettimada et al. (2018) found that plasma EVs from ART-treated HIV-infected individuals, which had enhanced oxidative stress markers and Notch4, activated pro-inflammatory feedback when introduced to THP-1 monocytes, leading to the upregulation of genes responsible for interferon responses. Sampey et al. (2016b) documented that extracellular vesicles released by HIV-1-infected individuals contain TAR miRNAs and HIV-1 TAR-RNA. These molecules activate the secretion of pro-inflammatory cytokines, TNF-a and IL-4, by stimulating components of the NF-kB pathway through their interaction with PKR and potentially TLRs. Kulkarni and Prasad (2017b) described that HIV-1 infected DCs derived extracellular vesicles comprised galectin-3 and fibronectin, which function in up-regulating the expression of pro-inflammatory cytokines and stimulating the p38/stat mechanism in T-cells. Conversely, by modulating T-cell receptor signaling and influencing the differentiation and proliferation of T cells, EVs can also suppress immune functions. T cell function is impaired by immunosuppressive molecules such as Nef protein in extracellular vesicles from HIV-infected cells, which contribute to immune evasion by the virus, affect the susceptibility of T cells to HIV infection and influence the persistence of the virus in infected individuals (Guha et al., 2019a). Novel HIV-1-derived microRNAs (miRNAs), specifically vmiR88 and vmiR99, are detectable in the bloodstream of individuals who are HIV-positive and can function independently of the inhibition of gene expression. These miRNAs act as ligands for the TLR8 signaling pathway in human macrophages, stimulating the release of TNFα (Tumor Necrosis Factor alpha). This process contributes to chronic immune activation observed in HIV-infected individuals (Bernard et al., 2014). Evs in human breast milk have the potential to rival HIV-1 in attachment to DC-SIGN on DCs, providing a protective effect against vertical transmission of HIV-1 (Naslund et al., 2014). EVs from TLR3-activated cells, including those from human brain microvascular endothelial cells and intestinal epithelial cells, contain antiviral elements capable of hindering HIV replication and stimulating the production of antiviral interferons, along with cellular HIV restriction factors (Sun et al., 2016).

### 3.3 The role of extracellular vesicles in HIV diagnosis

**FIGURE 5:**
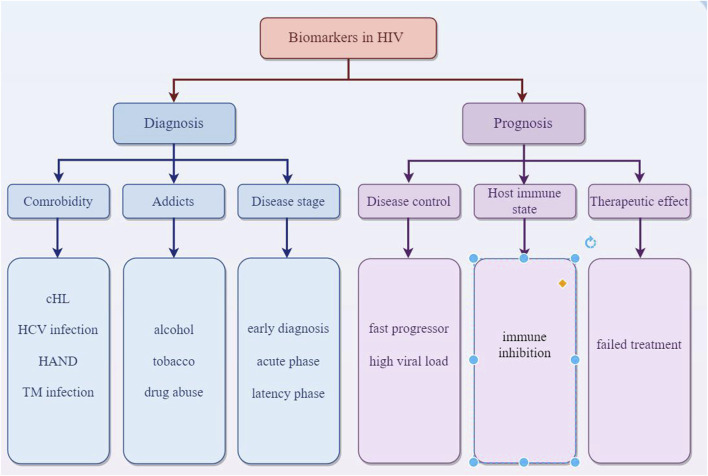
EVs act as biomarkers in diagnosis of prognosis of HIV.

EVs offer a promising avenue for HIV diagnosis due to their accessibility, their ability to carry HIV-specific markers, and the potential for early detection of the virus. EVs are abundant in various body fluids like blood, saliva, and urine. This makes them accessible for non-invasive diagnostic methods. In the case of HIV, EVs can carry viral RNA and proteins. Detecting these specific HIV components within EVs can provide evidence of HIV infection. EVs can interact with HIV particles or infected cells. This interaction can influence the disease progression and can be a marker for diagnosis. The content of EVs changes in response to HIV infection. This change in the molecular profile of EVs can serve as a biomarker for HIV. Modern diagnostic techniques like nanoparticle tracking analysis, flow cytometry, and mass spectrometry can analyze EVs for their size, quantity, and molecular content, aiding in early and accurate HIV diagnosis. Since EVs can carry HIV genetic material even in early infection stages, they might help in diagnosing HIV earlier than traditional methods. Ongoing research is focused on harnessing EVs for more efficient and less invasive HIV diagnostics, potentially improving early detection and monitoring of the disease. MiR-20a and miR-21 related to cHL in HIV-1-infected individuals could aid in early detection and treatment strategies for cHL in the context of HIV infection (Pulliam et al., 2019). Several biomarkers from plasma neuron derived EVs were recently reported to be elevated in general cognitive impairment (Khan et al., 2016; Kodidela et al., 2020a) and neurodegeneration (Hubert et al., 2015) in HIV. The astrocytic indicator GFAP and the neuronal marker L1CAM could serve as potential biomarkers for detecting neurological impairment associated with HIV infection (Hernández-Walias F. et al., 2020). Meanwhile, EVs hold potential as biomarkers for individuals with HIV who abuse drugs. Consumption of tobacco and alcohol can alter the pattern of chemokines and cytokines in EVs, contributing to the spread of disease and toxicity in those infected with HIV. Specifically, levels of IL-8 were found to be higher in HIV-positive individuals who consumed alcohol, whereas IL-10 levels were elevated in those infected with HIV who smoked (Kodidela et al., 2018; Kodidela et al., 2020b). Proteomic studies of plasma EVs from HIV-positive patients who regularly use tobacco and alcohol revealed changes in several proteins, such as alpha-2-macroglobulin, properdin, and hemopexin. These proteins in EVs may act as potential indicators of drug abuse in HIV-infected persons (Kodidela et al., 2020c). Cognitive impairment seen in HIV-associated neurological disease (HAND) may be induced by Nef in target cells and subsequently increasing expression and secretion of beta-amyloid (Aβ) and Aβ peptides (Mahfuz et al., 2016). Nicole et. examines circulating biomarkers, such as NF-L, sCD163, and sCD14, which are crucial for detecting alterations in the central nervous system (CNS) and signify immediate physiological changes in patients (Fernandes and Pulliam, 2021). There is a correlation between the increase of cerebrospinal fluid (CSF) extracellular vesicles and the neuronal injury biomarker NFL in individuals with HIV who are undergoing cART treatment and suffering from neurocognitive impairment (Guha et al., 2019b). miR-3162-3p is downregulated in the acute phase of HIV infection, making it a potential biomarker for new infections (Moghoofei et al., 2018). miR-146b-5p and miR-150 are examples of miRNAs that exhibit altered expression at different stages of HIV infection, serving as potential markers for disease progression (Munshi et al., 2014). Specifically, the levels of miR-31, miR-200c, miR-526a, miR-99a, and miR-503 may act as indicators for individuals who experience rapid progression of the disease (Munshi et al., 2014). Specifically, the levels of miR-31, miR-200c, miR-526a, miR-99a, and miR-503 may act as indicators for individuals who experience rapid progression of the disease (Sung and Rice, 2009). Duskova et al. (2013) identified that miR-19b, miR-146a, miR-615-3p, miR-382, miR-34a, miR-144, and miR-155, which regulate genes associated with the innate immune response and inflammation, were significantly increased in peripheral blood mononuclear cells (PBMCs) with the increased viral loading in the people infected with HIV-1. During the latency phase of HIV infection, the elevated level of miRNAs like miR-28, miR-125b, miR-150, miR-223, and miR-382 in infected cells contributes to the inhibition of virus production (Huang et al., 2007b). Additionally, miRNAs such as miR-17-5p, miR-20a, and miR-29a suppress viral replication during this phase (Patel et al., 2014). Rocca et al. observed a negative correlation between miR-29a levels and both HIV viral load and the extent of immunosuppression. Patients who had low miR-29a levels, especially those with CD4 counts below 350 cells/μL who did not respond to treatment, suggest that miR-29a could serve as a potential biomarker for identifying long-term survivors and assessing responses to ART. Furthermore, miR-29a may have the potential to predict disease prognosis and progression (Rosca et al., 2016). Additionally, four neuro-inflammation-related miRNAs (146a, 126, 21, and let-7a) were found to be elevated in HIV-infected individuals who use heroin (Wang et al., 2020b). The study examined the surface markers of EVs in patients coinfected with HIV and HCV, identifying a higher expression of markers such as HLA-DR/DP/DQ, CD81, and CD8. This suggests a link with the immune activation associated with individuals coinfected with HIV and HCV, highlighting the role of these miRNAs in modulating fatty-acid metabolism genes. This finding is significant as it relates to liver disease progression and the response to antiviral therapies in HIV/HCV coinfected patients (Martínez-González et al., 2020). Functional discrepancies between extracellular vesicles from HIV-infected and uninfected individuals could be used for early detection of HIV infection (Connor et al., 1998). Some certain miRNAs, particularly miR-192-5p, miR-194-5p, and miR-1246, were significantly upregulated in serum extracellular vesicles from AIDS patients with Talaromyces marneffei (TM) infection. The combination of these three miRNAs had an area under the curve (AUC) of 0.742, with a sensitivity of 56.8% and a specificity of 86.1% in predicting the presence of AIDS in conjunction with TM infection, which showed potential as non-invasive biomarkers for early diagnosis of AIDS in patients with TM infection (Ning et al., 2021) (Figure 5).

However, it is important to note that this is a rapidly evolving field, and continuous research is required to fully realize the diagnostic potential of EVs in HIV.

### 3.4 Potential of extracellular vesicles in HIV therapy

EVs are key players in the modulation of immune responses in the context of HIV infection. Their dual role in both activating and suppressing immune functions, their impact on T-cell modulation, and their potential in therapeutic and vaccine development make them a significant focus in HIV research and treatment strategies. MiR-192-5p, miR-194-5p, and miR-1246 are involved in important signaling pathways such as TGF-β, AMPK, cAMP, Wnt, and MAPK signaling pathways in HIV patient with Talaromyces marneffei (TM) concurrent infection (Ning et al., 2021). TAR RNA in EVs from HIV-1 infected cells could be a potential target for cancer therapeutics in HIV patient coinfected with cancer (Sharma, 2019). Targeting these novel HIV-1-derived miRNAs, specifically vmiR88 and vmiR99, could exhibit a potential therapeutic strategy for mitigating chronic immune activation and slowing the progression of AIDS (Naslund et al., 2014). Targeting the interaction between EVs and TIM-4 could be a potential strategy for inhibiting HIV-1 infection (Sims et al., 2017).

Monitoring the presence of circulating EVs and the microRNAs they carry is feasible and holds the potential to offer fresh insights into HIV-1 pathogenesis, disease progression, the associated inflammatory state, and the effectiveness of ART and immune activation reduction interventions (Lattanzi and Federico, 2012a). Certain microRNAs (miR-192, miR-144, miR-21), cytokines (IL-6, sCD14), and proteins have been independently linked to different aspects of HIV infection, making them innovative tools for predicting CD4 cell recovery and its trajectory with and after ART. miR-192, miR-144, IL-6 and sCD14 may be used to foresee the inadequate recovery of CD4 cells and its evolution after the commencement of ART (QIU et al., 2018a). Otherwise, Exosome-originated miR-21 was autonomously linked to the reduction of CD4 T cells in HIV-1-infected elite controllers (Wang et al., 2017). Additionally, the detection of specific HIV-1 related proteins in the urine of HIV-1 patients, along with neuron-derived extracellular vesicles (NDE) containing HMGB1, NF-L, and Aβ proteins, holds promise as potential biomarkers for cognitive impairment due to HIV-1 (Bhowmik and Chaubey, 2022a). IgM class antibodies demonstrate the ability to eliminate retroviral particles and cells infected with retroviruses through complement activation, even in the presence of complement-inhibiting proteins on the retroviral surface. This suggests that engineered IgM molecules targeted at specific retroviral epitopes could be beneficial in reducing retroviral infections, potentially eradicating some retroviral reservoirs (Gould et al., 2003). Scaffold Attachment Factor B-Like Transcription Modulator (SLTM) is found as a new factor in HIV-1 silencing and has a potential as a target for therapeutic interventions aimed at reactivating and eliminating latent HIV-1 reservoirs (Pedersen et al., 2022).

Additional *in vivo* investigations may lead to the potential development of vaccines for HIV-1 that are based on exosomes containing Nef (Lattanzi and Federico, 2012b). Extracellular vesicles have the potential to be used as delivery vehicles for antiretroviral drugs or other therapeutic agents aimed at modulating immune responses in HIV-infected individuals (QIU et al., 2018b). The ability of extracellular vesicles to carry antigens and stimulate immune responses makes them promising candidates for vaccine development against HIV. A increase in both number and functionality of CD8^+^ cytotoxic T lymphocytes and restoration of CTL functionality in HIV chronic infection is induced by the Gag-Texo vaccine in mTORC1 pathway, which should significantly influence the advancement of novel therapeutic vaccines for human immunodeficiency virus (HIV-1) infection (Akbay et al., 2020). They could be engineered to present HIV antigens to elicit a specific immune response. The composition of EVs in HIV-infected individuals could reflect the state of the immune system and serve as biomarkers for immune status or the efficacy of antiretroviral therapy.

## 4 Challenges and future perspectives

The exploration of EVs in HIV research presents a blend of challenges and opportunities, from technical and administrative hurdles to promising future directions and their potential in overcoming HIV drug resistance and side effects. One of the primary technical challenges is the isolation and characterization of EVs (Li et al., 2022; Wu et al., 2023; Zhang et al., 2023). Due to their small size and heterogeneity, separating EVs from other extracellular vesicles and cellular debris requires sophisticated and often expensive techniques.

There is a lack of standardization in EVs research. Different studies use varied methods for exosome isolation and analysis, making it difficult to compare results across studies. Key methods of specialized techniques for detecting and analyzing extracellular vesicles (EVs) in the context of HIV infection include ultracentrifugation coupled with density gradient separation for isolating EVs, immunoaffinity capture for high specificity, nanoparticle tracking analysis (NTA) for sizing and concentration, transmission electron microscopy (TEM) with immuno-gold labeling for high-resolution imaging, flow cytometry for high-throughput analysis, and molecular techniques like PCR and next-generation sequencing (NGS) for analyzing nucleic acid content. These methods, each with its advantages and limitations, are integral for a comprehensive analysis of EVs, offering insights into their molecular composition, concentration, and potential roles in HIV infection. The integration of these techniques is crucial for advancing our understanding of HIV-EV interactions and their implications for disease progression and potential therapeutic interventions. Administratively, securing funding and fostering multidisciplinary collaborations can be challenging. EVs research spans biology, immunology, virology, and nanotechnology, requiring coordinated efforts across these fields. Future research directions in exosome and HIV interaction should be focused on understanding of mechanisms, therapeutic agents, vaccine development and potential of EVs in overcoming HIV drug resistance and side effects. A key area of future research is to elucidate the detailed mechanisms of how EVs influence HIV infection and progression. This includes understanding how EVs interact with HIV particles and infected cells. Exploring the potential of EVs as vehicles for targeted drug delivery, especially for delivering antiretroviral drugs to specific cells or tissues affected by HIV. Investigating the role of EVs in vaccine development, particularly in presenting HIV antigens to the immune system to elicit a protective response. Unlike general antiretroviral drugs treatment, EVs have the potential to deliver drugs in a more targeted manner, potentially reducing the side effects associated with systemic administration of antiretroviral drugs and overcoming HIV drug resistance, a significant challenge in the current treatment of HIV/AIDS by delivering drugs directly to infected cells. EVs could be used to deliver gene-editing tools like CRISPR/Cas9 to infected cells (Bhowmik and Chaubey, 2022b), offering a novel approach to potentially cure HIV by excising the virus from the genome of infected cells.

In conclusion, while EVs research in the context of HIV faces various technical and administrative challenges, it also holds significant promise for future therapeutic and diagnostic advancements. Continued research and collaboration across disciplines are essential to fully realize the potential of EVs in understanding and treating HIV.
